# Sodium Reduction by Partial and Total Replacement of NaCl with KCl in Serbian White Brined Cheese

**DOI:** 10.3390/foods11030374

**Published:** 2022-01-27

**Authors:** Jelena Miocinovic, Zorana Miloradovic, Mira Radovanovic, Ivana Sredovic Ignjatovic, Ana Radulovic, Maciej Nastaj, Bartosz G. Sołowiej, Igor Tomasevic

**Affiliations:** 1 Department of Animal Source Food Technology, Faculty of Agriculture, University of Belgrade, Nemanjina 6, 11081 Belgrade, Serbia; jmiocin@agrif.bg.ac.rs (J.M.); zorana@agrif.bg.ac.rs (Z.M.); m.radovanovic@agrif.bg.ac.rs (M.R.); aradulovic@agrif.bg.ac.rs (A.R.); 2 Department of Chemistry and Biochemistry, Faculty of Agriculture, University of Belgrade, Nemanjina 6, 11081 Belgrade, Serbia; isredovic@agrif.bg.ac.rs; 3 Department of Dairy Technology and Functional Foods, Faculty of Food Sciences and Biotechnology, University of Life Sciences in Lublin, Skromna 8, 20-704 Lublin, Poland; mnasty@tlen.pl (M.N.); bartosz.solowiej@up.lublin.pl (B.G.S.)

**Keywords:** sodium reduction, white brined cheese, consumer acceptance, TPA, proteolysis

## Abstract

Cheese has been listed as one of four priority food groups intended for salt reduction reformulation. The present study aimed to investigate the possibility of producing Serbian white brined cheese (Homoljski Sir) with half of NaCl, three quarters of NaCl and all NaCl replaced with KCl (Na50, Na25 and Na0, respectively). Basic composition, proteolysis and texture profile parameters were monitored during 60 days of ripening. At the end of ripening, an acceptance test was conducted by untrained consumers (*N* = 46). According to the cluster analysis based on hedonic scores, three clusters emerged: male consumers (47.8%), agreeable consumers (30.4%) and highly educated female consumers (21.8%). Both partial and a total salt replacement had no effect on the course of proteolytical changes, the texture and basic composition during ripening. Female consumers did not accept any level of salt substitution, while male consumers showed dislike only for the Na0 cheese. Almost 80% of all consumers liked moderately-to-very-much the Na25 cheese variant. It implies that it is worth considering the production of cheese with 50–75% of NaCl replaced with KCl. The addition of natural flavoring and clear labeling of the sodium reduction should accompany the salt replacement strategy.

## 1. Introduction

Salt (NaCl) has a crucial impact on cheese quality and safety. It determines water activity, controls microbial growth and enzyme activity, and influences biochemical transformations during ripening. Salt affects the water-binding capacity of casein and thereby sets the rheological and textural characteristics and cooking properties of cheese [1]. The main positive effect of salt is the preservation and enhancement of cheese flavor [2]. The reduction or replacement of salt has numerous negative effects on cheese quality and safety, such as increased amount of bitter peptides, undesirably softened texture [3], excessive fermentation, growth of harmless non-starter bacteria [4], etc.

The tendency to keep high levels of salt in order to improve cheese quality and safety carries a health warning. It is well known that sodium intake is associated with an increased risk of numerous health problems [5]. Nevertheless, in many countries, daily salt intake significantly exceeds the recommended amount (less than 5 g of NaCl or 2 g of Na per day) [5]. Among the under-seventy population, excessive salt consumption is five times more common in central Asia and in central and eastern Europe than in western Europe [5].

The World Health Organization has listed salt reduction as one of the five priority interventions in the prevention and control of noncommunicable diseases [5]. Numerous salt reduction strategies and programs have been initiated throughout Europe. The salt reduction programs include multidisciplinary actions such as: public education, product reformulation and clear labeling of salt content in order to help consumers make informed decisions when purchasing food [6]. 

Cheese has been listed as one of four priority food groups intended for salt reduction reformulation [4,7]. Moreover, the most popular type in Serbia—the white brined cheese group (WBC, or pickled cheese), is generally characterized with high salt content (55–90 g·L^−1^ of salt-in-moisture S/M) [1,8,9]. Homoljski Sir is one of the known type of Serbian white brined cheeses and is characterized by protection of geographical origin on a national level. This cheese is produced in a specific region (Homolje mountain) from cow, goat or sheep milk. Homoljski Sir belongs to soft and full-fat cheeses with at least one month of ripening [10].

Two major sensory characteristics of white brined cheese are acidity and saltiness [9]. Although an alarmingly high salt/sodium content of WBC calls for technological intervention, to reduce the popular salty component of its taste would be a challenge to consumer acceptance. It has been reported that Serbian consumers of the 24-and-under age cohort prefer the salty variant of goat pickled cheese compared to the reduced salt variant [11]. In a study of consumer preferences regarding artisan cheese, only those of ages 55 and over were aware of the excessive saltiness of Serbian traditional cheeses [8]. The motivation to reduce sodium intake varies among different groups of the population, which certainly limits the effectiveness of salt reduction strategies. An alternative strategy proposed is to develop low sodium cheese sub-varieties of the existing cheeses. Such added-value products would target specific groups of consumers and aim to meet their particular needs [4,12].

In order to reformulate cheese technology in terms of salt reduction, two different approaches have been adopted: a simple reduction of NaCl and its partial replacement with KCl, CaCl_2_ or MgCl_2_, with or without additives/flavor intensifiers. Recently, the use of salt alternatives (micro crystal KCL, brown wrack seaweed, etc.) has also been reported as a very promising solution, but it is still at a concept stage [6]. 

Replacing a portion of NaCl with KCl (salt replacement in further text) is the most commonly used method for sodium reduction in various cheese types such as São João Portuguese cheese [13], feta cheese [14], Nabulsi cheese [15] and Turkish white cheese [1,16]. Although the method was studied by numerous authors over the course of the last few decades, and was reviewed recently [3,17,18], only limited data are available regarding the application of such reformulation on white brined cheeses [14,15,19,20,21]. Taking into account all three aspects of sodium content in cheese—health, cheese quality and safety, consumer acceptance—this study aimed to investigate the possibility of producing Serbian white brined cheese (Homoljski Sir) with half of NaCl, three quarters of NaCl and all NaCl replaced with KCl. The first objective was to characterize physico-chemical, textural and sensorial properties of this product, and to determine potential changes of compositional characteristics and ripening parameters caused by salt replacement. The second objective was to ascertain the segmentation of consumers based on their sensory acceptance of investigated cheeses, in order to identify potential focus groups for different strategies of sodium reduction. It would be beneficial not just for Homoljski Sir but also for other similar white brined cheeses that are characterized with very high salt content. The present study is the first attempt of replacement of NaCl with KCl in Serbian white brined cheeses.

## 2. Material and Methods

### 2.1. Cheese Manufacture and Sampling

Milk for cheese production was from a herd of 50 commercial Holstein cows. Cheese was produced in the EkoFil dairy plant (Petrovac na Mlavi, Serbia) during the months of March and April, following the process described in Table 1.

The manufacturing cycle was repeated twice, once in each month. Samples were collected at the beginning (1st–5th day), middle (30th day) and end (60th day) of ripening, to obtain data for basic composition, proteolysis and texture. At the end of ripening, samples were collected for macroelements and sensory analysis.

### 2.2. Basic Cheese Composition

Prior to analysis, the whole block of each cheese variant was ground and homogenized in order to obtain uniformity. Cheese composition was determined with standard methods [20]: a drying method at 102 ± 2 °C was used for moisture/dry matter content; the van Gulik method was used for fat content; protein content was determined by measuring nitrogen content (Kjeldahl method) and multiplying it by 6.38; chloride content was determined by the Volhard method; pH was measured in cheese slurry (10 g of cheese resolve in 10 mL of distilled water) with a pH-meter (Consort, Turnhout, Belgium). All analyses were conducted in triplicate.

### 2.3. Macroelements

Prior to determination of the content of macroelements, the homogenized sample (0.5 g) was placed in a PTFE vessel, followed by the addition of 4 cm^3^ 65% HNO_3_ and 1 cm^3^ 30% H_2_O_2_. The mixture was digested in a microwave oven (CEM Mars 5, Charlotte, NC, USA) at a temperature of 200 °C using a pressure controlled program. At first, pressure was raised to 1.36 atm/20 psi (3 min ramp) and held for 1 min. Afterwards, pressure was raised to 4.08 atm/60 psi during 8 min and held for 5 min. The sample was cooled to room temperature, topped up to 25 mL with deionized water and conserved at 4 °C until the analysis with ICP.

The content of macroelements Na, K, Mg and Ca was determined by ICP-OES, model iCAP 6500 Duo (Thermo Scientific, Gloucester, UK). The calibration curves were constructed by dilution of PE-CAL4-ASL-1 standard solution for Na, K, Mg and Ca. All measurements were performed in triplicate, and results are presented as mean value with standard deviation.

### 2.4. Proteolysis Assessment

The extent of proteolysis during ripening was assessed by analyzing the amounts of the water soluble nitrogen fraction (WSN), nitrogen fraction soluble in 5% phosphotungstic acid (PTA) and by urea polyacrylamide gel electrophoresis (Urea-PAGE).

In order to determine the WSN fraction, cheese extracts were prepared by the Kuchroo and Fox method [21], while PTA nitrogen compounds were extracted by the method of Stadhouders [22]. Ripening index RI was calculated as a percentage of WSN fraction in the total nitrogen content (TN). The content of PTA soluble nitrogen was also expressed as a percentage of TN, and will be denoted as PTA/TN.

The water insoluble cheese fraction was analyzed by Urea-PAGE, according to the Andrews method [23], using an Electrophoresis unit 20.5 × 10 cm Twin plate, TV200YK (Consort, Belgium), with the EV202 power supply run at 80 mA of constant current and at 300 V—the maximum voltage.

**Figure 1:**
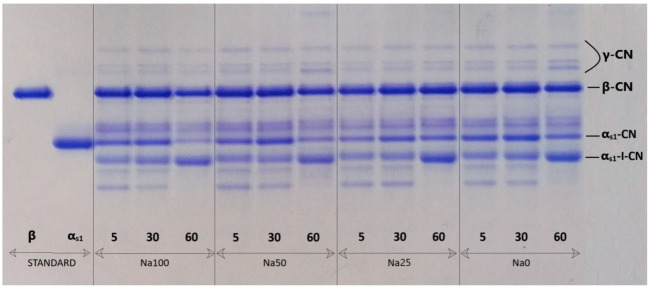
Urea polyacrylamide gel electrophoresis of Homoljski Sir with 100% NaCl and 0% KCl (Na100); 50% NaCl and 50% KCl (Na50); 25% NaCl and 75% KCl (Na25) and 0% NaCl and 100% KCl (Na0), at the 5th, 30th and 60th day of ripening. Other abbreviations are: CN—casein; STANDARD proteins: β—casein (β) and α—casein (α).

After the electrophoresis, gels were stained and de-stained following the procedure described in detail by Miloradovic et al. [24]. Residual casein fractions (β-CN and α_s1_-CN) and products of its degradation (γ1, γ2 and γ3 and α_s1_-I-CN) were identified (Figure 1). Relative quantification of protein band intensity was performed with Image J software. The area of bands β-CN, γ-CN, α_S1_-CN and α_S1_-I-CN (f24–199) for each cheese sample were expressed as a percentage of the total band area. The parameter representing the degree of β-casein degradation (β-CN index) was calculated as:

(1) $$\beta -\mathrm{CNindex}=\frac{\gamma 1+\gamma 2+\gamma 3}{\beta -\mathrm{CN}}$$

The degree of α_s1_-casein degradation (α_s1_-CN index) was calculated as:

(2) $$\alpha s1-\mathrm{CNindex}=\frac{\alpha s1-I-\mathrm{CN}}{\alpha s1-\mathrm{CN}+\alpha s1-I-\mathrm{CN}}$$

### 2.5. Texture Profile Analysis (TPA)

Texture profile analysis (TPA) was performed on each sampling day and six samples of each cheese variant were analyzed. Cheeses were sampled at 4 °C by cutting, with a sharp cylindrical cutter, to an equal-sized cylinder of 15 mm height and 15 mm in diameter. After cutting, the samples were immediately covered with a plastic wrap-adhesive membrane and left before testing for 30 min to equilibrate to room temperature. TPA parameters such as hardness, adhesiveness, springiness, chewiness, cohesiveness and resilience were measured using a TA.XTplus Texture Analyzer (Stable Micro Systems, Godalming, Surrey, UK) equipped with 5 kg loading cell and P/25 cylinder probe, and calculated from the force versus time curve, using the Exponent software provided by the same producer. The samples were compressed axially in two consecutive cycles with 33.3% deformation (5 mm) from the initial sample’s height, at 120 mm min_1 rate of force application.

### 2.6. Consumer Testing for Sensory Acceptance of Cheeses

Sensory testing was conducted after 60 days of cheese ripening. The consumer sample involved 46 volunteers, aged between 21 and 60. They consisted of personal contacts: colleagues, relatives and friends. Prior to sensory testing, demographic characteristics (gender, age and education) were collected.

The nine-point hedonic scale was used to test whether consumers liked cheese variants in terms of odor, flavor and texture, and whether they liked ’the product as a whole’. Score 1 was assigned to extreme dislike of cheese, while 9 was for extreme liking. Score 5 was neutral liking.

### 2.7. Statistical Analysis

Cheesemaking procedures were undertaken in duplicate and all analyses were conducted in triplicate. Salting conditions (the level of salt replacement) and ripening time were considered as fixed factors. Two-way ANOVA was conducted to determine the effect of those factors. One-way ANOVA was conducted for PTA/TN content and for macroelements (that were analyzed only at the end of ripening time). A subsequent post-hoc Tukey test was performed, with the level of significance of 0.05.

Hierarchical cluster analysis was conducted in order to define consumer profiles. Consumers (*N* = 46) were divided into three clusters on the basis of the hedonic test scores, using Ward’s clustering method and with squared Euclidean distance interval as a measure. Mann–Whitney U test was conducted in order to identify significant differences between clusters (*p* < 0.05).

IBM SPSS Statistics 21 was used for all statistical analysis.

## 3. Results and Discussion

### 3.1. Basic Composition

Ripening time had a significant effect on all basic compositional parameters (Table 2). Typically for this cheese group, pH decreased significantly at the beginning of ripening, causing an increase in dry matter content and, consequently, in protein and fat content. The same pattern was observed in studies of other WBC types [15,22].

Considering fat in dry matter FDM (>71%) and moisture on a fat-free basis MFFB (>50%), and according to regulative norms [23], all cheeses belong to the group of soft, full-fat cheeses.

It has been reported that salt replacement could cause alteration in the cheese fermentation process and thus promote a pH drop in cheeses with lower sodium content [3]. However, in the present study, salt replacement did not have a significant effect on any basic compositional parameter.

The present study confirmed what has already been suggested; that when sodium reduction is carried out, it is easier to maintain cheese characteristics as unchanged if the initial content of NaCl is high [4].

### 3.2. Macroelements

Salt replacement was verified by the analysis of a gradual decrease in Na content followed by an increase in K content (Table 1). Regarding health, applied reformulations are highly positive, especially if arterial pressure is considered, because both low sodium and high potassium intake content have beneficial effects. The recommended proportion of Na and K intake is set at 0.5–0.6 [4], so it could be assumed that the Na25 cheese has the optimal Na/K proportion (0.53).

The results for all analyzed macroelements in Na100 (control cheese variant) are comparable to the results for those in feta cheese, which has the highest amount of sodium among all 21 listed cheese types [24]. When half of the NaCl was replaced with KCl, the sodium content of Homoljski Sir became comparable to brie, cheddar or Stilton. When three quarters of NaCl were replaced, the sodium content approached the amount found in Emmental or cottage cheese. In the Na0, where total replacement was carried out, sodium content was similar to fromage frais, the unsalted cheese variant [24]. As expected, and due to the added KCl, K content in all experimental variants (except the Na100) was higher than in the aforementioned cheeses. The amount of Mg and Ca was not significantly affected by the type of salt used.

In order to be declared “low sodium”, cheese should contain less than 280 mg/100 g of sodium. Since the control variant (currently produced in EkoFil) contains more than five times that salt content, more than 75% of NaCl needs to be reduced or replaced before the cheese could be labeled as “low sodium“ [25].

### 3.3. Proteolysis Assessment

Proteolytical changes of Homoljski Sir were assessed via the ripening index (RI) and casein degradation indexes (α-CN index and β-CN index). Furthermore, the PTA/TN ratio was examined on the 60th day of ripening in order to check if there was a significant effect of salt replacement on the depth of proteolysis. Results from Figure 1 and Table 3 show that the pattern of proteolytical changes was typical for WBC, affecting α_s__1_-casein and WSN after two months of ripening, while β-casein stayed intact.

During two months of ripening, sodium reduction had no significant effect on proteolysis in Homoljski Sir, just as it has been reported for cheeses of similar type [15,21]. The results showed that there was a steady increase of PTA/TN with a decrease of sodium. The increase was not significant, but it would be reasonable to expect that a ripening period longer than two months would result in more intensive peptide production, especially if the high amounts of NaCl were replaced with KCl. Similar results were reported by Ayyash and Shah [15] for 3–5 month-ripened WBC. 

The insignificant influence of salt replacement on proteolysis could be regarded as positive, because, in some cases, salt reduction increases the ratio of proteinase/peptidase activity. It results in a bitter taste because bitter peptides tend to form excessively and to degrade insufficiently [3]. 

### 3.4. Textural Properties of Cheese

Ripening had a significant effect on all textural parameters (Table 4). During the first month of ripening, hardness and chewiness increased significantly, and they decreased during the second month, but never reached the initial level for either of the mentioned parameters. As in the study that investigated textural changes of the Iranian ultra-filtered feta cheese type, the increase of the aforementioned parameters could be attributed to a pH drop and subsequent whey migration from cheese to brine [2]. During the second month of ripening, hardness and chewiness decreased—proteolytical changes that significantly affected αs1 casein (Table 3). Such changes were in line with the existing literature [3,11]. Resilience, cohesiveness and springiness exhibited the opposite trend—they decreased after 30 days and then increased after 60. 

There was no significant effect of either salt replacement or of the interaction between ripening and salt replacement on the texture of cheeses. It has been reported that the 50:50% mixture of NaCl and KCl does not cause textural changes [3]. The present study showed that for white brined cheeses, which have high initial NaCl content, textural changes did not occur after two months of ripening, even in the case of total salt replacement.

A more pronounced proteolytic degradation, a usual cause of texture alteration in cheeses with reduced sodium, could lead to undesirable softness in some varieties [3]. This was obviously not the case with Homoljski Sir that was subjected to 60 days of ripening.

### 3.5. Consumer Testing for Sensory Acceptance of Cheeses

As explained, and presented in Table 5, three clusters of consumers were identified, based on the scores they gave to the four cheese variants during hedonic testing.

Cluster 1 differed significantly from Cluster 3 in terms of gender: it had a higher percentage of male consumers. Therefore, Cluster 1 (47.8%) will be called male consumers. Cluster 3 had a significantly higher percentage of consumers with university degrees and more females compared to Cluster 2, so Cluster 3 (21.8%) will be labeled *highly educated female consumers.*

Table 6 presents hedonic test scores given by consumers divided in clusters. It is important to notice that the clusters did not differ in the acceptance of the Na100 variant. The scores for overall liking were between 7 (like moderately) and 8 (like very much). Considering the high level of salt in the Na100 cheese (≈68 g L^−1^ of salt in moisture S/M), it confirms what has already been reported [9]: that consumers in Serbia like and tolerate a high amount of salt in cheese either because they are unaware of the risk it creates or because they accept it. When lots of food with high salt content is consumed, people get used to an excessive salt intake and tend to like it more [4]. 

*Highly educated female consumers* disliked Na50 cheese (50% NaCl replaced with KCl) and gave it significantly lower scores than the rest of the consumers. The study conducted in Belgium has revealed that sodium intake is higher among men than among women [26]. The present study suggests that *highly educated female consumers* show higher sensitivity to a distinctly different, slightly bitter and metal-like flavor sensation that the addition of KCl brings to cheese [2,17]. It is yet to be investigated whether this is related to their gender or to their education, or maybe to a combination of both. Unlike those given by *highly educated female consumers*, hedonic scores given by *male consumers* dropped below 5 (in the range of ‘dislike’) only when NaCl was totally replaced with KCl. Consumers from Cluster 2 accepted with moderate liking even the variant that had NaCl totally replaced with KCl. It could be concluded that in the present sample, Cluster 2 (30.4%) consisted of *highly agreeable consumers.*

Guinee and O’Kennedy [27] reported discrepancies among studies that tested consumer liking for different salt replacement ratios. According to these authors, less than 50% of NaCl could be substituted with KCl, without negative effect on sensorial acceptability of cheeses. It is generally agreed that a replacement of only 10–25% is not detectable organoleptically and does not impair technological properties of cheeses [4]. Differences that exist in literature are due either to the type of cheese analyzed, or to the initial amount of NaCl. However, the results of the present study showed that discrepancies also existed among groups of consumers of different genders and of different education levels.

An important finding of the present study was that even when 75% of NaCl was replaced with KCl, nearly 80% of consumers still showed moderate-to-very-much liking. Since no physico-chemical or ripening parameter was altered in two months of ripening, it is worth considering the development of a special low sodium sub-type of Homoljski Sir, or of any other type of Serbian white brined cheeses. The development strategy could be to mask potential off-flavors with some natural additives or flavoring ingredients. Moreover, it would be important to clearly inform consumers about the reduction of sodium content and the health benefits attached to it, which, as already reported, increases their interest in such products [4,28].

Since highly educated female consumers showed high sensitivity to KCl-originated off-flavors, it could be suggested that gradual step-by-step reduction of salt, proposed by some authors [12,17], should also be considered for Homoljski Sir and all other over-salted Serbian cheeses.

## 4. Conclusions

This study revealed that even 75% replacement of NaCl with KCl was not sufficient to obtain “low sodium” (less than 280 mg/100 g of sodium) for two-month-ripened Homoljski Sir. From the perspective of technological properties, even with a total salt replacement, neither the course of proteolytical changes nor the texture and basic composition was altered in two months of cheese ripening.

The moderate-to-very-much acceptance of Na100 cheese by all consumers, regardless of the cluster membership, indicated that they were used to over-salted white brined cheeses. Different groups of consumers showed different sensitivity to KCl off-flavors. The most sensitive were *highly educated women* and it needs to be investigated whether education or gender was the key factor. Over 80% of consumers showed moderate-to-very-much liking for cheese with 75% of NaCl replaced.

Taking into account all the findings of the present study, we suggest that the Serbian cheese industry should take responsibility for and make a contribution to salt reduction in over-salted white brined cheese varieties. In general, a gradual step-by-step reduction of salt is recommended. For the investigated Homoljski Sir, it would be worth considering the production of cheese with 50–75% of NaCl being replaced with KCl. The addition of natural flavoring additives could be used for masking potential off-flavors. The salt replacement strategy could be accompanied by clear labeling of the sodium reduction and the health benefits attached to that.

It is important to note that the reduction in health problems related to high sodium intake is not likely unless, together with salt reduction, proper physical activities, weight watching and other healthy habits are also practiced.

## Figures and Tables

**Table 1:** Production process for Serbian white brined cheese—Homoljski Sir.

Time Lapse	Manufacturing Procedures	
Pre-processing of milk	Standardization of fat to 3.5–3.6%, pasteurization (72 °C/20 s) and cooling (32 ± 1 °C)	
0 min	Addition of starter culture ^a^ until the acidity reaches 7.4–7.6° SH	
30 min	Addition of CaCl_2_ and rennet ^b^ (0,16 g/10 L)	
1 h 20 min	Cutting of the gel into 5 cm cubes and resting	
1 h 25 min	Transfer of curd to rectangular press	
1 h 30 min	Draining	
2 h 30 min	Pressing	
6 h	Cutting the curd into blocks 10 × 10 cm (250 g), and dry salting at surface, with 4 different salt mixtures and packing into plastic containers	Salt mixtures:
		Na100 (100% NaCl)
		Na50 (50% NaCl and 50% KCl)
		Na25 (25% NaCl and 75% KCl)
		Na0 (100% KCl)
6 h 30 min	Storing at room temperature	
30 h 30 min	Ripening in 4 different brine solutions (12%) for 60 days at 16–18 °C	Brine solutions:
		Na100 (100% NaCl)
		Na50 (50% NaCl and 50% KCl)
		Na25 (25% NaCl and 75% KCl)
		Na0 (100% KCl)

^a^ Lactoferm MST4-10U (Biochem s.r.l., Bologna, Italy) containing *Lactococcus lactis* subsp. *Lactis*, *Lactococcus lactis* subsp. *cremoris* and *Streptococcus salivarius* subsp. *thermophilus.* ^b^ Maxiren 1800 (DSM, Heerlen, Netherlands).

**Table 2:** Basic composition and macroelements of white brined cheese manufactured with different ratios of NaCl and KCl after 1, 30 and 60 days of ripening.

Parameter Days				Cheese Variants			
			Sig *	Na100	Na50	Na25	Na0
pH							
1			c	5.36 ± 0.06	5.35 ± 0.06	5.33 ± 0.12	5.38 ± 0.16
30			b	4.69 ± 0.02	4.57 ± 0.04	4.59 ± 0.02	4.53 ± 0.10
60			a	4.32 ± 0.02	4.29 ± 0.02	4.36 ± 0.08	4.29 ± 0.02
DM (%)							
1			a	39.27 ± 1.11	38.93 ± 0.22	38.63 ± 0.34	38.61 ± 0.40
30			b	47.24 ± 2.67	46.95 ± 0.74	47.85 ± 2.73	48.14 ± 1.34
60			b	46.95 ± 0.34	47.32 ± 0.14	48.64 ± 2.35	46.21 ± 1.38
MF (%)							
1			a	20.00 ± 0.00	21.50 ± 0.00	20.50 ± 0.58	20.00 ± 0.00
30			b	27.25 ± 0.87	27.50 ± 1.73	30.50 ± 2.89	33.50 ± 7.50
60			b	28.50 ± 0.58	28.50 ± 0.58	28.50 ± 0.58	28.50 ± 0.57
TP (%)							
1			a	13.10 ± 1.06	13.73 ± 0.49	13.71 ± 0.92	13.05 ± 0.76
30			b	16.31 ± 1.32	16.10 ± 0.68	16.29 ± 2.21	16.67 ± 1.04
60			b	17.31 ± 0.77	15.88 ± 0.81	17.22 ± 0.99	16.44 ± 1.27
60		Cl^−^ (%)		2.05 ± 0,44	1.87 ± 0.20	1.84 ± 0.14	1.59 ± 0.17
Macroelements:							
60	Na (mg/100 g)			1542.0 ± 146.0 ^a^	750.5 ± 75.6 ^b^	408.5 ± 17.7 ^c^	39.3 ± 3.54 ^d^
60	K (mg/100 g)			113.0 ± 11.3 ^a^	365.5 ± 74.2 ^b^	768.5 ± 36.1 ^c^	1266.0 ± 219.2 ^d^
60	Ca (mg/100 g)			297.0 ± 52.74	236.0 ± 2.84	270.0 ± 13.6	292.0 ± 24.8
60	Mg (mg/100 g)			10.43 ± 2.79	10.10 ± 2.84	13.35 ± 0.92	10.28 ± 2.58

* Different letters (a, b, c, d) indicate significant differences considering ripening time, for *p* < 0.05. Cheese variants: Na100—cheese with 100% NaCl and 0% KCl; Na50—cheese with 50% NaCl and 50% KCl; Na25—cheese with 25% NaCl and 75% KCl; Na0—cheese with 0% NaCl and 100% KCl. Parameter: DM—dry matter; MF—milk fat; TP—total protein.

**Table 3:** Proteolysis parameters of white brined cheese manufactured with different ratios of NaCl and KCl after 1, 30 and 60 days of ripening.

Parameter Days		Cheese Variants			
	Sig *	Na100	Na50	Na25	Na0
α_s__1_-index					
4	a	0.50 ± 0.02	0.53 ± 0.04	0.55 ± 0.05	0.52 ± 0.04
30	a	0.50 ± 0.01	0.51 ± 0.02	0.54 ± 0.03	0.53 ± 0.03
60	b	0.61 ± 0.04	0.61 ± 0.03	0.60 ± 0.03	0.58 ± 0.06
β-CN index					
4	a	0.76 ± 0.17	0.74 ± 0.13	0.76 ± 0.13	0.75 ± 0.10
30	a	0.73 ± 0.17	0.73 ± 0.13	0.73 ± 0.10	0.72 ± 0.09
60	a	0.78 ± 0.16	0.87 ± 0.05	0.79 ± 0.01	0.76 ± 0.13
RI					
1	a	8.15 ± 1.10	7.76 ± 0.77	6.61 ± 1.98	8.07 ± 0.87
30	a	9.68 ± 1.19	8.09 ± 1.48	9.33 ± 0.65	7.77 ± 1.89
60	b	10.58 ± 2.10	10.69 ± 1.44	11.43 ± 0.78	11.77 ± 0.50
PTA/TN					
60		0.81 ± 0.07	0.78 ± 0.19	0.81 ± 0.03	1.24 ± 0.31

* Different letters (a, b) indicate significant differences considering ripening time, for *p* < 0.05. Cheese variants: Na100—cheese with 100% NaCl and 0% KCl; Na50—cheese with 50% NaCl and 50% KCl; Na25—cheese with 25% NaCl and 75% KCl; Na0—cheese with 0% NaCl and 100% KCl. PTA/TN—percentage of PTA-soluble nitrogen in total nitrogen; RI–ripening index (water soluble nitrogen in total nitrogen).

**Table 4:** Textural parameter of white brined cheese manufactured with different ratios of NaCl and KCl after 1, 30 and 60 days of ripening.

Parameter Days		Cheese Variants			
	Sig *	Na100	Na50	Na25	Na0
Hardness (g)					
1	a	344.5 ± 24.2	339.4 ± 28.1	307.9 ± 16.4	273.3 ± 28.9
30	c	1543.2 ± 35.0	1528.9 ± 82.8	1640.8 ± 123.5	1667.3 ± 269.3
60	b	1065.5 ± 202.0	1206.1 ± 114.5	996.6 ± 17.9	1085.4 ± 102.4
Adhesiveness (g s)					
1	c	22.2 ± 0.4	22.8 ± 0.8	20.6 ± 2.4	20.9 ± 1.4
30	b	10.6 ± 2.1	10.6 ± 2.8	8.9 ± 1.1	8.1 ± 2.3
60	a	4.1 ± 1.3	3.6 ± 1.7	3.9 ± 2.3	4.1 ± 1.8
Springiness (mm)					
1	b	0.93 ± 0.04	0.97 ± 0.03	0.95 ± 0.01	0.97 ± 0.03
30	a	0.79 ± 0.05	0.80 ± 0.05	0.87 ± 0.08	0.76 ± 0.05
60	b	0.97 ± 0.04	0.93 ± 0.07	0.93 ± 0.08	0.91 ± 0.03
Cohesiveness					
1	b	0.83 ± 0.02	0.82 ± 0.02	0.83 ± 0.01	0.83 ± 0.03
30	a	0.46 ± 0.05	0.47 ± 0.11	0.52 ± 0.10	0.54 ± 0.10
60	a	0.50 ± 0.06	0.44 ± 0.02	0.49 ± 0.03	0.46 ± 0.02
Chewiness					
1	a	265.0 ± 15.4	262.4 ± 23.7	216.5 ± 14.0	214.8 ± 21.3
30	c	496.5 ± 19.0	480.1 ± 13.1	502.4 ± 61.1	492.2 ± 59.3
60	b	266.6 ± 19.3	242.3 ± 29.3	289.6 ± 47.9	278.3 ± 48.4
Resilience					
1	b	0.46 ± 0.02	0.46 ± 0.01	0.47 ± 0.01	0.45 ± 0.02
30	a	0.16 ± 0.03	0.19 ± 0.04	0.18 ± 0.04	0.19 ± 0.05
60	a	0.21 ± 0.01	0.19 ± 0.01	0.21 ± 0.03	0.21 ± 0.03

* Different letters (a, b, c) indicate significant differences considering ripening time, for *p* < 0.05. Cheese variants: Na100—cheese with 100% NaCl and 0% KCl; Na50—cheese with 50% NaCl and 50% KCl; Na25—cheese with 25% NaCl and 75% KCl; Na0—cheese with 0% NaCl and 100% KCl.

**Table 5:** Structure of a cluster membership.

		**Overall (100%)**	**Cluster 1** **(47.8%)**	**Cluster 2** **(30.4%)**	**Cluster 3 (21.8%)**
Gender (%)	Female	41.3	22.7	50.0	70.0
	Male	58.7	77.3	50.0	30.0
Age (%)	21–40	52.2	45.5	57.1	60.0
	41–60	47.8	54.5	42.9	40.0
Education (%)	Elementary/High school	63.1	63.6	85.7	30.0
	University: BSc, MSc, PhD	36.9	36.4	14.3	70.0

**Table 6:** Consumer scores for white brined cheeses salted with different ratios of NaCl and KCl.

Hedonic Attributes		Consumers (*N* = 46)		
		Cluster 1 (47.8%)	Cluster 2 (30.4%)	Cluster 3 (21.8%)
Na100	OVERALL	7.45 ± 1,26	7.50 ± 1.56	7.10 ± 2.02
	ODOR	7.05 ± 1.86	6.71 ± 2.46	7.40 ± 1.78
	TASTE	7.59 ± 1.65	6.93 ± 2.73	6.70 ± 1.83
	TEXTURE	7.64 ± 1.18	7.64 ± 1.22	7.00 ± 2.11
Na50	OVERALL	7.32 ± 1.52 ^b^	7.14 ± 1.66 ^ab^	5.40 ± 2.32 ^a^
	ODOR	7.32 ± 1.70 ^b^	7.71 ± 1.27 ^b^	5.10 ± 1.97 ^a^
	TASTE	7.68 ± 1.49 ^b^	7.07 ± 1.77 ^ab^	4.60 ± 2.99 ^a^
	TEXTURE	7.45 ± 1.65 ^b^	7.71 ± 1.27 ^b^	5.00 ± 2.00 ^a^
Na25	OVERALL	7.36 ± 1.18 ^b^	8.07 ± 0.73 ^b^	3.50 ± 2.01 ^a^
	ODOR	7.09 ± 1.72 ^b^	7.79 ± 1.05 ^b^	4.60 ± 1.90 ^a^
	TASTE	7.00 ± 1.72 ^b^	7.93 ± 1.59 ^b^	3.60 ± 1.84 ^a^
	TEXTURE	7.23 ± 1.60 ^b^	8.00 ± 1.24 ^b^	3.90 ± 2.02 ^a^
Na0	OVERALL	4.86 ± 1.91 ^a^	7.07 ± 1.49 ^b^	3.60 ± 2.50 ^a^
	ODOR	4.82 ± 1.65 ^a^	7.50 ± 1.09 ^b^	3.30 ± 2.00 ^a^
	TASTE	3.50 ± 2.09 ^a^	7.29 ± 1.38 ^b^	2.80 ± 2.04 ^a^
	TEXTURE	5.45 ± 1.65 ^a^	7.79 ± 0.89 ^b^	4.10 ± 0.99 ^a^

Different letters (a, b) indicate significant differences considering cluster membership, for *p* < 0.05. Cheese variants: Na100—cheese with 100% NaCl and 0% KCl; Na50—cheese with 50% NaCl and 50% KCl; Na25—cheese with 25% NaCl and 75% KCl; Na0—cheese with 0% NaCl and 100% KCl.

## Data Availability

The data presented in this study are available on request from the corresponding author.
